# Bioconverted Fruit Extract of Akebia Quinata Exhibits Anti-Obesity Effects in High-Fat Diet-Induced Obese Rats

**DOI:** 10.3390/nu14214683

**Published:** 2022-11-05

**Authors:** Seul Gi Lee, Eunbi Lee, Jongbeom Chae, Jin Soo Kim, Han-Saem Lee, Yu-Mi Lim, Jai-Hyun So, Dongyup Hahn, Ju-Ock Nam

**Affiliations:** 1 Department of Food Science and Biotechnology, Kyungpook National University, Daegu 41566, Korea; 2 National Development Institute of Korean Medicine, Gyeongsan-si 38540, Gyeongsangbuk-do, Korea; 3 Institute of Agricultural Science and Technology, Kyungpook National University, Daegu 41404, Korea; 4 Research Institute of Tailored Food Technology, Kyungpook National University, Daegu 41566, Korea

**Keywords:** 3T3-L1 adipocytes, *Akebia quinate*, anti-adipogenic, bioconversion, chocolate vine, obesity

## Abstract

*Akebia quinata*, commonly called chocolate vine, has various bioactivities, including antioxidant and anti-obesity properties. However, the anti-obesity effects of bioconverted extracts of *A. quinate* have not been examined. In this study, *A. quinata* fruit extracts was bioconverted using the enzyme isolated from the soybean paste fungi *Aspergillus kawachii.* To determine whether the bioconversion process could influence the anti-obesity effects of *A. quinata* fruit extracts, we employed 3T3-L1 adipocytes and HFD-induced obese rats. We observed that the bioconverted fruit extract of *A. quinata* (BFE) afforded anti-obesity effects, which were stronger than that for the non-bioconverted fruit extract (FE) of *A. quinata*. In 3T3-L1 adipocytes, treatment with BFE at concentrations of 20 and 40 μg reduced intracellular lipids by 74.8 (*p* < 0.05) and 54.9% (*p* < 0.01), respectively, without inducing cytotoxicity in preadipocytes. Moreover, the oral administration of BFE at the concentration of 300 mg/kg/day significantly reduced body and adipose tissue weights (*p* < 0.01) in HFD-induced obese rats. Plasma cholesterol values were reduced, whereas HDL was increased in BFE receiving rats. Although FE could exert anti-obesity effects, BFE supplementation induced more robust effects than FE. These results could be attributed to the bioconversion-induced alteration of bioactive compound content within the extract.

## 1. Introduction

Obesity is a serious public health challenge that can increase risk factors for developing various diseases, such as hyperlipidemia, diabetes, hypertension, and certain cancers [1]. Since 1980, the global prevalence of overweight and obesity has increased two-fold [2]. At a molecular level, strategies to treat and prevent obesity include the inhibition of preadipocyte proliferation and differentiation [3], suppressing lipogenesis [4], and promoting lipolysis [5]. In the present study, we focused on preadipocyte differentiation and lipogenesis inhibition. Adipogenic differentiation occurs through a series of stages, in which multipotent stem cells (MSCs) are transformed to mature adipocytes [6]. Initially, MSCs are converted into preadipocytes for adipogenic differentiation, followed by clonal expansion, CCAAT-enhancer-binding protein beta (C/EBPβ) expression, and accumulation of lipid droplets [7]. Subsequently, peroxisome proliferator-activated receptor-gamma (PPARγ) and CCAAT-enhancer-binding protein beta (C/EBPα) are expressed, resulting in lipogenesis, during which the lipid droplet size increases [8]. Lipogenic enzymes, such as lipoprotein lipase (LPL) and fatty acid-binding proteins, are generated during this process [9]. In addition, mature adipocytes, generated through differentiation in vivo, secrete adipokines, such as adiponectin, to reduce vascular inflammation or increase insulin sensitivity [10].

Herbal extracts have been attracted attention as potential anti-obesity agents. Compared with pharmaceutical agents, herbal extracts are generally considered safe, as they are well-accepted and induce few serious side effects [11]. One other hands, herbal medicines may produce adverse effects that can range from mild to severe, such as allergic reactions, headaches, stomach upsets, and hepatotoxicity [12]. Several crude herbal extracts and medicines have been reported to provide health benefits, such as anti-oxidative, anti-obesity, and anti-diabetic effects [13,14]. Bioconversion biotechnology, also referred to as biotransformation, using microorganism-derived enzymes could potentially improve the efficiency and application of herbal extracts [15]. Previous studies reported Microbial biotransformation induced improved the anti-oxidant capacity of *Pericarpium Citri Reticulatae* (PCR) extract and also suggested that the biotransformation may be useful in other herbal extracts [16]. Additionally, biotransformation enhanced the production of bioactive compound resveratrol in peanut skin [17].

*Akebia quinata*, (*A. quinata)* commonly called chocolate vine, is used in traditional medicine in East Asia and is also consumed as food [18,19]. *A. quinata* is pharmacopoeial species approved in the European Union as plant raw material and has been reported to contain various bioactive compounds, such as saponins, oleanolic acid, hederagenin, and akequintoside D. [20,21]. Previous studies reported that the extracts of *A. quinata* fruit and leaves exerted anti-obesity effects in vivo and in vitro [18,22]. However, the effects of bioconverted *A. quinata* remain unexplored.

In the present study, we aimed to investigate how the bioconversion process impacts the anti-obesity effects mediated by bioconverted fruit extracts of *A. quinata* (BFE) when compared with the non-bioconverted fruit extracts of *A. quinata* (FE) in high-fat diet (HFD)-induced obese rats and 3T3-L1 adipocytes. We hypothesize that BFE could help ameliorate obesity more significantly than FE by regulating the expression of PPARγ, C/EBPα, and adiponectin.

## 2. Materials and Methods

### 2.1. Sample Preparation

FE and BFE were obtained from the National Development Institute of Korean Medicine. The extraction process was performed as previously described [23,24]. Briefly, *A. quinata* was refluxed with 70% MeOH, filtered through filter paper, concentrated using a rotary evaporator, bioconverted using the enzyme isolated from the soybean paste fungi *Aspergillus kawachii*, and then fractionated sequentially in butanol (BuOH) [25]. The obtained extracts were stored at −20 °C until further use.

### 2.2. Animals and Diet

Six-week-old male Sprague–Dawley rats were purchased from Hyochang Science (Daegu, Korea), housed in cages, and acclimatized for 1 week under a 12 h light/dark cycle at 25–30 °C. After acclimatization, the rats were randomly assigned and housed separately to the following groups (*n* = 6 per group): (1) normal diet (ND), (2) HFD, (3 and 4) HFD supplemented with FE and BFE at 150 mg/kg/day (henceforth referred to as FE 150 and BFE 150, respectively), and (5 and 6) HFD supplemented with FE and BFE at 300 mg/kg/day (henceforth referred to as FE 300 and BFE 300, respectively). High-fat diet (HFD) with 60% fat (D12492; 5.2 kcal/g) was purchased from Research Diets Inc. (New Brunswick, NJ, USA). During the predetermined study period, FE and BFE were orally administered daily. Food and water were provided ad libitum for 4 weeks, following which blood and adipose tissue samples were collected for further analysis. Animal experiments were approved by the Institutional Animal Care Committee of Kyungpook National University, Daegu, South Korea (approval number: KNU 2017-0084).

### 2.3. Blood Biochemical Tests

Collected blood specimens were centrifuged at 3000× *g* for 15 min to obtain the plasma supernatant. The plasma levels of aspartate aminotransferase (AST), alanine aminotransferase (ALT), high-density lipoprotein (HDL), cholesterol (CHOL), blood urea nitrogen (BUN), creatinine (CRE), alkaline phosphatase (ALP), protein, and glucose were determined using an Olympus AU 400 (Olympus Optical, Tokyo, Japan) analyzer, according to the manufacturer’s instructions.

### 2.4. Cell Culture and Differentiation

3T3-L1 mouse preadipocyte cells were purchased from the Korea Cell Line Bank (KCLB, Seoul, Korea) and cultured in Dulbecco’s modified Eagle’s medium (DMEM; Gibco, Grand Island, NY, USA), supplemented with 10% bovine calf serum (Gibco). To induce cell differentiation, 3T3-L1 adipocytes were cultured to post-confluence (designated as day 0) for 2 days, and the medium was replaced with DMEM containing 10% fetal bovine serum (FBS; Gibco) and MDI solution (0.5 mM IBMX, 1 μM DEXA, 0.125 mM indomethacin, and 10 μg/mL insulin) for 2 days. The cells were maintained in a medium supplemented with 10% FBS and 10 μg/mL insulin for 4 days. To assess the anti-adipogenic effects of FE and BFE, cells undergoing differentiation were treated with FE or BFE for 24 h and then removed. Treated and control cells were subsequently induced for differentiation into adipocytes.

### 2.5. MTT (3-[4,5-Dimethylthiazo-2-y1]-2,5-Diphenytetrazolium Bromide) Assay

Briefly, 3T3-L1 preadipocytes were seeded in a 96-well-plate and treated with BFE or FE for 24 h. At the end of the treatment period, MTT solution was added to each well, and plates were incubated for 3 h. Formazan crystals were dissolved in isopropyl alcohol (Duksan Pure Chemicals, Seoul, Korea).

### 2.6. Oil Red O Staining and Triglyceride (TG) Assay

Oil Red O staining and TG assays were performed as described previously [26]. At end-stage differentiation, fixed cells were stained with 0.4% Oil Red O, washed with distilled water, and photographed under a microscope at 200× magnification. The intracellular TG content was measured using a TG quantification kit (Bio Vision, CA, USA), according to the manufacturer’s instructions. The colorimetric intensity was determined at 570 nm.

### 2.7. Real-Time Quantitative Polymerase Chain Reaction (qPCR)

Total RNA was isolated using RNAiso Plus reagent (TaKaRa Bio, Shiga, Japan). An equal amount of total RNA was used to synthesize complementary DNA using the Prime-Script RT Reagent Kit (TaKaRa Bio), according to the manufacturer’s protocol. To assess the anti-adipogenic effect of FE and BFE, the mRNA expression levels of 3T3-L1 adipocytes and rat tissue-derived samples were analyzed using end-point PCR and qPCR, respectively. qPCR was performed using SYBR Green (Toyobo, Osaka, Japan) in conjunction with an iCycler iQ™ Real-Time PCR Detection System (Bio-Rad Laboratories, Hercules, CA, USA). The expression levels of RNA isolated from mouse 3T3-L1 adipocytes and rat tissues were normalized to β-actin and GAPDH, respectively. All primers were synthesized by Macrogen (Seoul, Korea) and primer sequences are listed in Table 1.

### 2.8. Western Blot Analysis

Western blotting was performed as described in a previous report [27]. Briefly, cells were harvested and lysed in RIPA buffer. Total proteins were separated by 10% sodium dodecyl sulfate-polyacrylamide gel electrophoresis (SDS-PAGE) and transferred onto nitrocellulose membranes. The membranes were blocked using 5% non-fat skim milk and incubated with the following primary antibodies: PPARγ, adiponectin (both from Abcam, Cambridge, UK), C/EBPα (Cell Signaling Technology, Beverly, MA, USA), and β-actin (Santa Cruz Biotechnology, CA, USA).

### 2.9. Histology Analysis

Epididymal white adipose tissue (eWAT) and the liver were fixed with 4% paraformaldehyde and embedded in paraffin. Then, 5–7-μm-thick tissue sections were stained with hematoxylin and eosin. The adipocyte size (area μm^2^) was measured using three rats per group with ImageJ software (National Institutes of Health, Bethesda, MD, USA).

### 2.10. Statistical Analysis

Statistical analyses were performed by One-way ANOVA using SPSS ver.20.0 (SPSS Inc., Chicago, IL, USA). *p* values less than 0.05 were considered significant.

## 3. Results

### 3.1. Effects of FE and BFE on Differentiation and Lipid Accumulation in 3T3-L1 Preadipocytes

**Figure 1:**
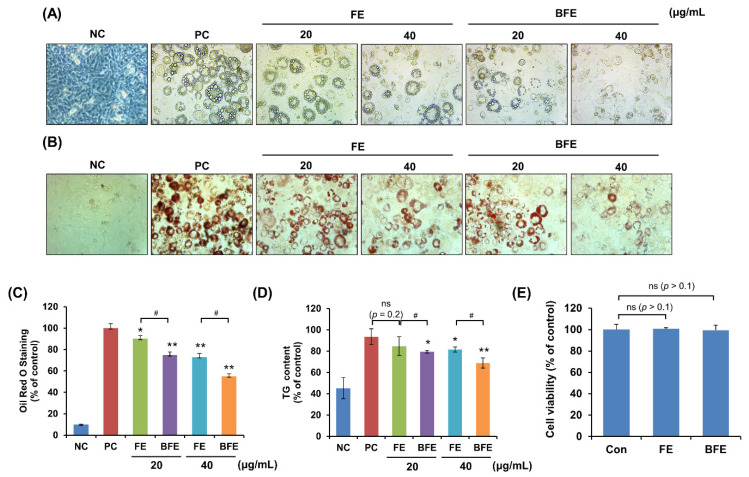
Effects of BFE and FE on lipid accumulation and triglyceride content during 3T3-L1 adipocyte differentiation without preadipocyte cytotoxicity. 3T3-L1 adipocytes were treated in differentiation media and BFE and FE at 20 and 40 μg/mL, respectively, for 8 days. (**A**) Cells were photographed immediately after 8 days of differentiation under different extract treatment conditions. (**B**,**C**) Lipid accumulation was measured by staining with Oil Red O solution, and absorbance was measured at 495 nm. (**D**) The intracellular TG content was measured using a TG assay kit, and absorbance was measured at 570 nm. Preadipocytes were used as negative controls (NC), and fully differentiated adipocytes without extract were used as positive controls (PC). (**E**) Effect of BFE and FE on the cell viability of 3T3-L1 preadipocytes. * *p* < 0.05 and ** *p* < 0.01 compared to control group. # *p* < 0.05 compared to the corresponding FE treated group. NS is defined as not statistically significant. PC, positive control; BFE, bioconverted fruit extract of *Akebia quinate*; FE, non-bioconverted fruit extract of *Akebia quinate*.

First, we investigated the anti-differentiation effect of FE and the differentiation of 3T3-L1 preadipocytes into mature adipocytes. Following treatment of 3T3-L1 adipocytes with different concentrations of FE and BFE, we compared the adipogenic capacity of treated and untreated cells. Compared to the differentiated group without treatment (Positive control; PC), treatment with FE and BFE inhibited the differentiation of 3T3-L1 adipocytes in a concentration-dependent manner (Figure 1A). The intracellular lipid and TG content of FE- and BFE-treated cells was reduced (Figure 1B–D). In particular, BFE exhibited stronger inhibitory activity against adipocyte differentiation and lipid accumulation than FE. To confirm whether the anti-differentiation effects of FE and BFE were caused by cell death or the inhibition of proliferation, we examined the effect of FE and BFE on preadipocyte viability. Treatment with 40 μg/mL FE or BFE for 24 h did not affect the viability of preadipocytes (Figure 1E). Therefore, we used a concentration of 0–40 μg/mL in subsequent experiments. Collectively, these results indicated that the anti-differentiation activity of BFE was enhanced following the bioconversion process.

### 3.2. Effects of FE and BFE on Protein Expression of Adipogenesis-Related Genes

**Figure 2:**
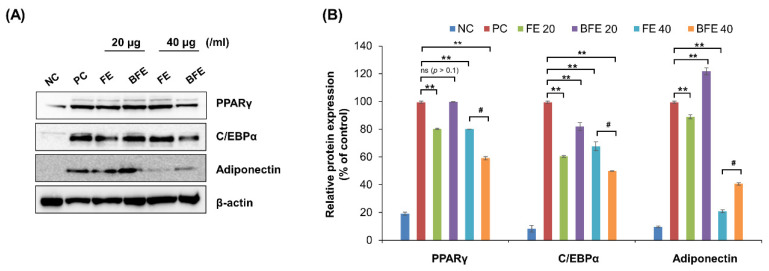
FE and BFE modulate protein expression levels of adipogenesis-related genes in 3T3-L1 adipocytes. 3T3-L1 preadipocytes cells were treated with FE or BFE at indicated concentrations for 24 h and induced for complete differentiation of adipocytes. (**A**) Representative images of Western blot results. (**B**) The signal intensities were quantified using ImageJ software. Each experiment was performed in triplicate. Bars represent the mean ± standard deviation (SD). ** *p* < 0.01 compared to control group. # *p* < 0.05 compared to the corresponding FE treated group. NS is defined as not statistically significant. BFE, bioconverted fruit extract of *Akebia quinate*; C/EBPβ, CCAAT-enhancer-binding protein beta; FE, non-bioconverted fruit extract of *Akebia quinate*; NC, negative control; PC, positive control; PPARγ, peroxisome proliferator-activated receptor-gamma.

To elucidate the mechanisms underlying the anti-differentiation effects of FE and BFE, we confirmed the protein expression of adipogenesis-related genes. Treatment with 40 μg/mL FE and BFE markedly suppressed the protein expression levels of PPARγ, C/EBPα, and adiponectin compared with that in untreated cells (Figure 2A,B). These findings suggested that FE and BFE inhibit adipogenesis via PPARγ signaling.

### 3.3. Effects of FE and BFE on HFD-Induced Obese Rats

**Figure 3:**
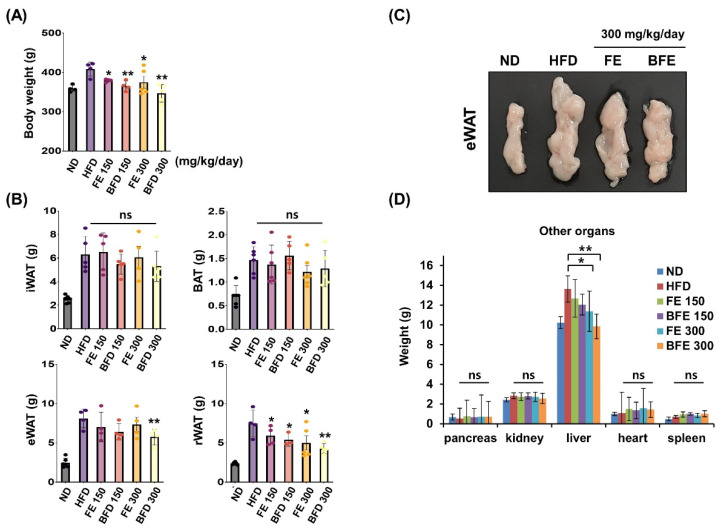
Effects of FE and BFE on body weight gain and tissue mass in HFD-induced obese rats. ND groups (*n* = 7) were fed a normal diet, HFD groups (*n* = 7) were fed HFD, and FE 150, BFE 150, FE 300, and BFE 300 groups (*n* = 7) were fed HFD plus FE or BFE at the indicated concentration. (**A**) Body weight was measured at the end of the study period. (**B**) Adipose tissue weight was separately measured. (**C**) Changes in eWAT size by diet type. (**D**) The weight of other organs was measured. Significant difference from the HFD group, ** *p* < 0.01 and * *p* < 0.05. Bar graphs represent the mean ± standard deviation (SD) from seven individual rats per group. NS is defined as not statistically significant. BFE, bioconverted fruit extract of *Akebia quinate*; eWAT, epididymal white adipose tissue; FE, non-bioconverted fruit extract of *Akebia quinate*; HFD, high-fat diet; ND, normal diet.

FE and BFE inhibited adipocyte differentiation and lipid accumulation, suggesting that FE and BFE could protect against HFD-induced obesity. Accordingly, we examined whether FE- and BFE-supplemented diets afforded anti-obesity effects in HFD-induced obese rats. After 4 weeks of oral administration, the FE 150 and BFE 150 groups exhibited body weights of 363.3 ± 4.6 and 352.8 ± 9.1 g, respectively (Figure 3A). The body weights of the animals in the FE 300 and BFE 300 groups were 362.0 ± 32.1 and 339.4 ± 22.1 g, respectively, while those of the HFD group reached 398.9 ± 6.6 g. Compared with the HFD group, the retroperitoneal white adipose tissue (rWAT) weight was significantly reduced in all experimental groups supplemented with FE and BFE (Figure 3B). In contrast, only the BFE 300 group demonstrated a significant decrease in eWAT weight (Figure 3B,C). Considering other organs, no difference was observed between the control and FE or BFE groups, except for the liver (Figure 3D). The reduced liver weight may be related to altered liver steatosis in the FE and BFE groups. These results demonstrated that FE- and BFE-supplemented diets could prevent HFD-induced excessive weight gain in a concentration-dependent manner. Interestingly, this preventive effect was more robust with BFE supplementation than with FE supplementation.

#### 3.3.1. Effects of FE and BFE on Feed Intake and Efficiency

**Figure 4:**
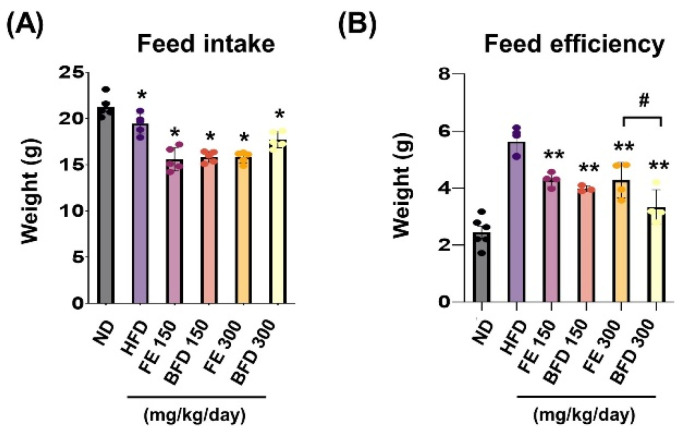
Effects of FE and BFE on feed intake and efficiency. Feed intake (**A**) was recorded daily, and the feed efficiency (**B**) ratio was calculated using the following formula: Body weight gain (g)/Amount of feed fed (g) × 100. Significant difference from the HFD ** *p* < 0.01 and * *p* < 0.05. # *p* < 0.05 compared to the corresponding FE treated group. Bar graphs represent the mean ± standard deviation (SD) from seven individual rats per group. BFE, bioconverted fruit extract of *Akebia quinate*; FE, non-bioconverted fruit extract of *Akebia quinate*; HFD, high-fat diet.

To confirm whether the suppression of body weight gain could be related to changes in appetite, we examined the influence of FE and BFE on feed intake and efficiency. We noted that feed intake and efficiency were lower in the FE and BFE groups than in the control group, regardless of concentration (Figure 4A). Notably, the BFE 300 group exhibited the lowest feed efficiency, even when compared with the FE group at the same concentration (Figure 4B). This result suggested that BFE treatment might be associated with high energy expenditure and fast metabolic process. Although we did not measure the caloric content of the stomach, these results offer indirect evidence that BFE could inhibit the caloric content of ingested food.

#### 3.3.2. Effects of FE and BFE on HFD-Induced Liver Steatosis and Adiposity

**Figure 5:**
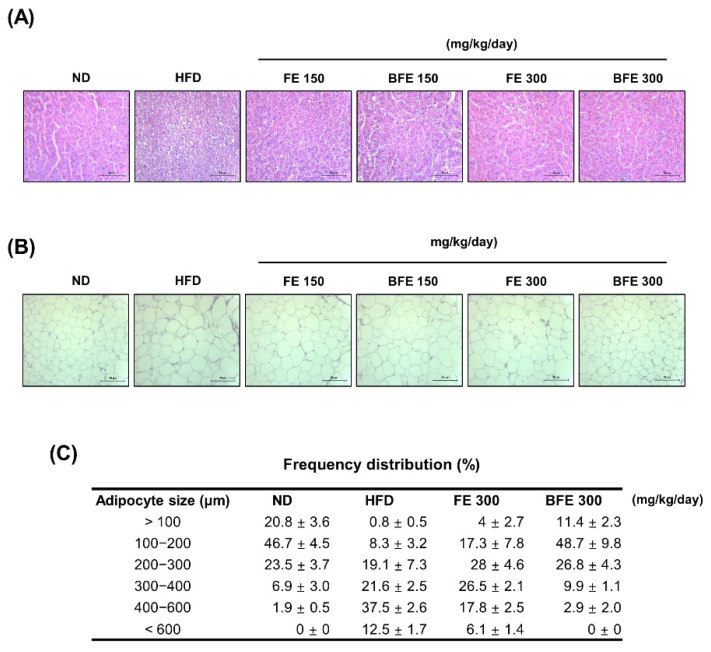
FE and BFE suppress lipid deposition in the liver and reduce adipocyte size in adipose tissues. Representative images of the liver (**A**) and eWAT (**B**) tissues stained with hematoxylin-eosin at 40× magnification (bar = 50 μm). Adipocyte size (**C**) in three rats per group was measured using ImageJ software. BFE, bioconverted fruit extract of *Akebia quinate*; FE, non-bioconverted fruit extract of *Akebia quinate*; HFD, high-fat diet; ND, normal diet.

Hepatic steatosis and adipocyte hypertrophy are common metabolic complications associated with obesity [28]. To examine whether FE and BFE could suppress HFD-induced hepatic steatosis and adipocyte hypertrophy, we performed a histopathological analysis of the liver and adipose tissue. Compared with the HFD group, lipid deposition in the liver was reduced in the FE and BFE groups in a dose-dependent manner (Figure 5A). On examining adipose tissue, the adipocyte size was decreased in the FE 150–300 and BFE 150–300 groups compared with the HFD group (Figure 5B,C). The adipocyte size in the BFE 300 group was comparable with that in the ND group. These results indicated that FE and BFE could alleviate lipid deposition in the liver and eWAT.

#### 3.3.3. Effects of FE and BFE on Plasma Lipid Concentration

**Figure 6:**
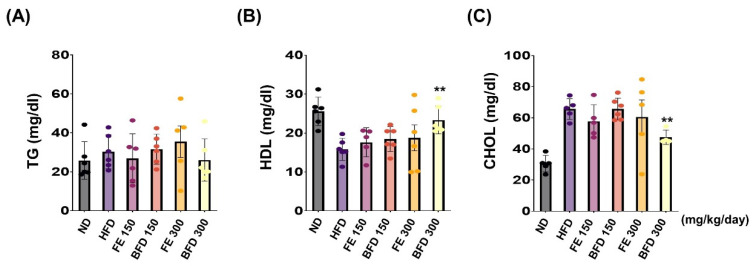
FE and BFE reduce plasma lipid concentration. (**A**–**C**) Blood plasma was collected to measure the TG, HDL, and CHOL. Significant difference from the HFD group, ** *p* < 0.01. Bar graphs represent the mean ± standard deviation (SD) from five individual rats per group. BFE, bioconverted fruit extract of *Akebia quinate*; FE, non-bioconverted fruit extract of *Akebia quinate*; HFD, high-fat diet; ND, normal diet.

Next, we investigated the effects of FE and BFE on plasma lipid concentration. Interestingly, we observed no significant differences between the FE 150–300/BFE 150 and HFD group. Only the BFE 300 group exhibited a reduction in the plasma CHOL content, and there was no significant difference documented in other groups (Figure 6A). Unexpectedly, TG content was not significantly different between groups (Figure 6A). On the other hands, the total HDL and CHOL content were significantly higher and lower, respectively, in the BFE 300 group than in the HFD group (Figure 6B,C). Furthermore, the levels of nephrotoxicity and hepatotoxicity markers, including albumin, ALP, AST, BUN, CRE, and total protein, did not significantly differ between FE, BFE, and control groups (Table S1). Collectively, these results suggested that BFE, unlike FE, could effectively improve HFD-induced dyslipidemia in rats.

### 3.4. Effects of FE and BFE on the Expression of Adipogenesis-Related Genes in Adipose Tissues

**Figure 7:**
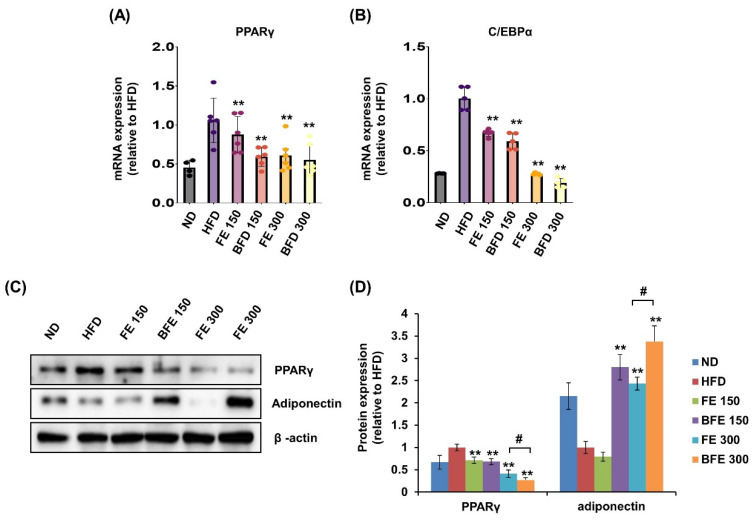
FE and BFE modulate the mRNA and protein expression in adipose tissues. (**A**) mRNA expression of PPARγ in rWAT. (**B**) mRNA expression of C/EBPα in rWAT. (**C**) Protein expression of PPARγ and adiponectin in rWAT. (**D**) Significant difference from the HFD group, ** *p* < 0.01. # *p* < 0.05 compared to the corresponding FE treated group. Bar graphs represent the mean ± standard deviation (SD) from seven individual rats per group. BFE, bioconverted fruit extract of *Akebia quinate*; C/EBPα, CCAAT-enhancer-binding protein beta; FE, non-bioconverted fruit extract of *Akebia quinate*; HFD, high-fat diet; ND, normal diet; PPARγ, peroxisome proliferator-activated receptor-gamma; rWAT, retroperitoneal white adipose tissue.

Visceral (including epididymal, mesenteric, and retroperitoneal) and subcutaneous (including inguinal and anterior subcutaneous) white adipose tissues are known to differ morphologically and functionally [29]. These differences may contribute to increased morbidity in patients with visceral obesity. In the present study, we examined the effects of FE and BFE on PPARγ expression in rWAT. In rWAT, the mRNA expression of PPARγ was reduced in the BFE 150, FE 150, and BFE 300 groups compared with the HFD group (Figure 7A). Likewise, the mRNA expression of C/EBPα was reduced in all experimental groups (Figure 7B). Similar to the mRNA expression, the protein expression levels of PPARγ were reduced in all experimental groups, with significantly lower expression detected in the BFE 300 group than in the FE 300 group (Figure 7C,D). In contrast to PPARγ levels, adiponectin expression was dramatically increased in the BFE 150, FE 300, and BFE 300 groups compared with the HFD group, with significantly higher expression noted in the BFE 300 group than in the FE 300 group. The pattern of adiponectin expression completely differed between in vivo (Figure 7C,D) and in vitro studies (Figure 2A,B). Adiponectin is an adipocyte-specific secretory protein that plays a major role in regulating insulin resistance and exerts anti-obesity effects [30]. Adiponectin is considered an obesity protection factor, as patients with obesity exhibit lower adiponectin concentrations than healthy subjects, whereas suppressed adiponectin expression is considered an anti-adipogenic marker in vitro [10,31]. Taken together, our data suggested that FE and BFE prevented HFD-induced obesity by inhibiting adipocyte differentiation and lipogenesis, which was potentially mediated via the PPARγ signaling pathway.

## 4. Discussion

Bioconversion, also known as biotransformation, may provide a strategy to enhance the pharmacological effects of natural extracts by modifying and generating the structure of bioactive compounds [32]. Previous studies have been implicated that bioconversion improved the anti-inflammatory, anti-cancer, and anti-angiogenic effects of herbal medicine, possible through enriching several compounds containing phenolic acid and flavonoid [23,33].

In the present study, we revealed that the *Aspergillus kawachii*-mediated bioconversion of A. quinata, previously known to exhibit anti-obesity effects, could afford improved anti-obesity effects. The FE and BFE treatment suppressed the differentiation and lipid accumulation in adipocytes. More importantly, BFE exerted significantly strong effects in most results compared to FE.

Mechanistically, we found that FE and BFE regulated the PPARγ signaling pathway. PPARγ is a key regulator of adipocyte differentiation and subsequently controls the secretion of adipokines [34]. Compared with FE at the same concentrations, BFE strongly reduced the expression levels of PPARγ, C/EBPα, and adiponectin in 3T3-L1 adipocytes. This result means that BFE inhibited lipogenesis via PPARγ pathway. Inconsistently with in vitro results, the adiponectin expression was significantly increased in BFE-treated rats in a dose-dependent manner. Indeed, adiponectin is reduced in obese patients and plays an important role in insulin sensitivity [35]. Additionally, although adiponectin is mainly secreted from adipocytes, it also is produced by other cell types including endothelial and muscle cells [36,37]. However, using an in vitro culture system, adiponectin was only released from adipocytes following BFE treatment. A corollary of the results above is that BFE treatment might contribute to not only the adipogenesis of adipocytes but also adipose metabolism including the differentiation and activation of other cell types.

We then explored the anti-obesity effect in HFD-induced obese rats to confirm the effect of bioconversion in vivo. BFE administration dose-dependently reduced eWAT and rWAT weights, suggesting a superior effect to FE treatment. In addition, BFE could significantly reduce liver weight when compared with FE. Accordingly, to confirm the effect of BFE and FE on HFD-induced liver steatosis and adiposity, the lipid droplet size in the liver and eWAT was compared. Treatment with BFE significantly reduced the average lipid droplet size. These results suggest that BFE is more effective than FE in inhibiting lipid deposition and lipid steatosis in the liver. Furthermore, these results suggest the possibility that BFE may be helpful in the treatment of non-alcoholic fatty liver disease (NAFLD).

We assumed that BFE and FE would also affect HFD-induced dyslipidemia. Plasma CHOL and HDL levels were measured after FE and BFE treatment. Not surprisingly, we found that only BFE 300 reduced plasma CHOL and increased HDL. These results suggest that both FE and BFE have anti-obesity effects, but, unlike FE, BFE has an effect of improving the metabolic profile of HFD-induced obese rats, suggesting the possibility of treating dyslipidemia with BFE. Future studies are needed to confirm the upstream regulator of the BFE-mediated PPARγ pathway, as well as the expression of lipolysis-related genes, which would support the development of BFE for anti-obesity therapy. If a more detailed mechanism of anti-obesity effect related to BFE is revealed and the expression of lipolysis-related gene is added to a further study, the possibility that BFE can be used as a treatment for obesity will increase. In addition, we must point out that specific modifications of a bioactive compound in BFE were not defined in the present study. Thus, the details of the chemical modification by bioconversion process in *A. quinata* extract need to be further investigated.

## 5. Conclusions

In conclusion, we demonstrated the anti-obesity effects of FE and BFE in vitro and in vivo using 3T3-L1 adipocytes and an HFD-induced obese rat model, respectively. Notably, the anti-obesity effects of BFE might be more effective than FE. Therefore, these results indicate that the bioconversion process of *A. quinata* might have potential benefits in terms of improving the anti-obesity effects, ameliorating body weight gain, adipose hypertrophy, hepatic steatosis, and the regulation of gene expression related to adipogenesis. Overall, these findings suggest that BFE, which is more effective than FE, can be developed as an effective material for preventing or treating obesity.

## Figures and Tables

**Table 1:** Sequences and accession numbers of primers used for qPCR.

Gene Name	Accession No.		Sequence
**Mouse**			
Adiponectin	NM_009605	Forward	5′- ACCTACGACCAGTATCAGGAAAAG-3′
		Reverse	3′- ACTAAGCTGAAAGTGTGTCGACTG-5′
C/EBPα	NM_001287523	Forward	5′- TTACAACAGGCCAGGTTTCC-3′
		Reverse	3′- GGCTGGCGACATACAGATCA-5′
LPL	NM_008509	Forward	5′- TCCTCTGACATTTGCAGGTCTATC-3′
		Reverse	3′- TCACGCCTTTCATAACACAT-5′
PPARγ	AB644275	Forward	5′- TTTTCAAGGGTGCCAGTTTC-3′
		Reverse	3′- AATCCTTGGCCCTCTGAGAT-5′
β-actin	EF095208	Forward	5′- GACAACGGCTCCGGCATGTGCAAAG-3′
		Reverse	3′- TTCACGGTTGGCCTTAGGGTTCAG-5′
**Rat**			
PPARγ	NM_013124.3	Forward	5′- CTTGGCCATATTTATAGCTGTCATTATT-3′
		Reverse	3′- GTGAAGCCCATCGAGGACA-5′
GAPDH	NM_017008.4	Forward	5′- TCTGACATGCCGCCTGGAGAA-3′
		Reverse	3′- TCATGGCCTACATGGCCTCCA-5′

## Data Availability

The data presented in this study are available on request from the corresponding author.

## Supplementary Materials

The following supporting information can be downloaded at: https://www.mdpi.com/article/10.3390/nu14214683/s1, Table S1: General characteristics of rats fed a normal or high-fat diet with or without FE and BFE.

## References

[1] Blüher M. (2019). Obesity: Global epidemiology and pathogenesis. Nat. Rev. Endocrinol..

[2] Afshin A., Forouzanfar M.H., Reitsma M.B., Sur P., Estep K., Lee A., Marczak L., Mokdad A.H., Moradi-Lakeh M., Naghavi M. (2017). Health Effects of Overweight and Obesity in 195 Countries over 25 Years. N. Engl. J. Med..

[3] Karschner V.A. (2010). Post-Transcriptional Regulation of mRNA Metabolism during Differentiation of 3T3-L1 Cells: Role of HuR.

[4] Ameer F., Scandiuzzi L., Hasnain S., Kalbacher H., Zaidi N. (2014). De novo lipogenesis in health and disease. Metab. Clin. Exp..

[5] Duncan R.E., Ahmadian M., Jaworski K., Sarkadi-Nagy E., Sul H.S. (2007). Regulation of lipolysis in adipocytes. Annu. Rev. Nutr..

[6] Ambele M.A., Dhanraj P., Giles R. (2020). Adipogenesis: A Complex Interplay of Multiple Molecular Determinants and Pathways. Int. J. Mol. Sci..

[7] Cheng G., Raza S.H.A., Khan R., Wang H., Shater A.F., Mohammedsaleh Z.M., Wang L., Tian Y., Long F., Zan L. (2021). C/EBPβ converts bovine fibroblasts to adipocytes without hormone cocktail induction. Electron. J. Biotechnol..

[8] Siersbaek R., Nielsen R., Mandrup S. (2010). PPARgamma in adipocyte differentiation and metabolism--novel insights from genome-wide studies. FEBS Lett..

[9] Niemelä S., Miettinen S., Sarkanen J., Ashammakhi N. (2008). Adipose tissue and adipocyte differentiation: Molecular and cellular aspects and tissue engineering applications. J. Tissue Eng. Regen. Med..

[10] Adamczak M., Wiȩcek A., Funahashi T., Chudek J., Kokot F., Matsuzawa Y. (2003). Decreased plasma adiponectin concentration in patients with essential hypertension. Am. J. Hypertens..

[11] Ekor M. (2014). The growing use of herbal medicines: Issues relating to adverse reactions and challenges in monitoring safety. Front. Pharmacol..

[12] Posadzki P., Watson L.K., Ernst E. (2013). Adverse effects of herbal medicines: An overview of systematic reviews. Clin. Med. (Lond.).

[13] Said O., Fulder S., Khalil K., Azaizeh H., Kassis E., Saad B. (2008). Maintaining a physiological blood glucose level with ‘glucolevel’, a combination of four anti-diabetes plants used in the traditional arab herbal medicine. Evid. Based Complement. Altern. Med..

[14] Bahmani M., Eftekhari Z., Saki K., Fazeli-Moghadam E., Jelodari M., Rafieian-Kopaei M. (2016). Obesity Phytotherapy: Review of Native Herbs Used in Traditional Medicine for Obesity. Evid. Based Complement. Altern. Med..

[15] Hussain A., Bose S., Wang J.-H., Yadav M.K., Mahajan G.B., Kim H. (2016). Fermentation, a feasible strategy for enhancing bioactivity of herbal medicines. Food Res. Int..

[16] Wang F., Chen L., Chen S., Chen H., Liu Y. (2021). Microbial biotransformation of Pericarpium Citri Reticulatae (PCR) by Aspergillus niger and effects on antioxidant activity. Food Sci. Nutr..

[17] Jin S., Gao M., Kong W., Yang B., Kuang H., Yang B., Fu Y., Cheng Y., Li H. (2020). Enhanced and sustainable pretreatment for bioconversion and extraction of resveratrol from peanut skin using ultrasound-assisted surfactant aqueous system with microbial consortia immobilized on cellulose. 3 Biotech..

[18] Sung Y.Y., Kim D.S., Kim H.K. (2015). Akebia quinata extract exerts anti-obesity and hypolipidemic effects in high-fat diet-fed mice and 3T3-L1 adipocytes. J. Ethnopharmacol..

[19] Lee D., Lee J.S., Sezirahiga J. (2020). Bioactive Phytochemicals Isolated from Akebia quinata Enhances Glucose-Stimulated Insulin Secretion by Inducing PDX-1. Plants.

[20] Maciąg D., Dobrowolska E., Sharafan M., Ekiert H., Tomczyk M., Szopa A. (2021). Akebia quinata and Akebia trifoliata—A review of phytochemical composition, ethnopharmacological approaches and biological studies. J. Ethnopharmacol..

[21] Kim G.-J., Chung K.-H., Lee K.J., An J.H. (2018). Ormosanine from Akebia quinata suppresses ethanol-induced inflammation and apoptosis and activates antioxidants via the mitogen activated protein kinase signaling pathway. J. Funct. Foods.

[22] Jeon Y.S., You Y.Y., Jun W.J. (2014). Anti-obesity Effects of Extracts from Young Akebia quinata D. Leaves. J. Korean Soc. Food Sci. Nutr..

[23] Lee S.G., Kim J.S., Lee H.S., Lim Y.M., So J.H., Hahn D., Ha Y.S., Nam J.O. (2017). Bioconverted Orostachys japonicas Extracts Suppress Angiogenic Activity of Ms-1 Endothelial Cells. Int. J. Mol. Sci..

[24] Kim M.-A., Lee H.-S., Chon S.-H., Park J.-E., Lim Y.-M., Kim E.-J., Son E.-K., Kim S.-J., So J.-H. (2019). Bioconversion of Gentiana scabra Bunge increases the anti-inflammatory effect in RAW 264.7 cells via MAP kinases and NF-κB pathway. J. Appl. Biol. Chem..

[25] Yang E.-J., Kim S.-I., Park S.-Y., Bang H.-Y., Jeong J.H., So J.-H., Rhee I.-K., Song K.-S. (2012). Fermentation enhances the in vitro antioxidative effect of onion (Allium cepa) via an increase in quercetin content. Food Chem. Toxicol..

[26] Lee S.G., Lee Y.J., Jang M.H., Kwon T.R., Nam J.O. (2017). Panax ginseng Leaf Extracts Exert Anti-Obesity Effects in High-Fat Diet-Induced Obese Rats. Nutrients.

[27] Lee S.G., Taeg K.K., Nam J.O. (2017). Silibinin Inhibits Adipogenesis and Induces Apoptosis in 3T3-L1 Adipocytes. Microbiol. Biotechnol. Lett..

[28] Gao M., Ma Y., Liu D. (2015). High-fat diet-induced adiposity, adipose inflammation, hepatic steatosis and hyperinsulinemia in outbred CD-1 mice. PLoS ONE.

[29] Wajchenberg B., Giannella-Neto D., Da Silva M., Santos R. (2002). Depot-specific hormonal characteristics of subcutaneous and visceral adipose tissue and their relation to the metabolic syndrome. Horm. Metab. Res..

[30] Poirier B., Bidouard J.P., Cadrouvele C., Marniquet X., Staels B., O’connor S., Janiak P., Herbert J.M. (2005). The anti-obesity effect of rimonabant is associated with an improved serum lipid profile. Diabetes Obes. Metab..

[31] Kajimoto K., Naraba H., Iwai N. (2006). MicroRNA and 3T3-L1 pre-adipocyte differentiation. Rna.

[32] Fang X., Du M., Liu T., Fang Q.a., Liao Z., Zhong Q., Chen J., Meng X., Zhou S., Wang J. (2019). Changes in the biotransformation of green tea catechins induced by different carbon and nitrogen sources in Aspergillus niger RAF106. Front. Microbiol..

[33] Song X., Wang L., Fan D. (2022). Insights into Recent Studies on Biotransformation and Pharmacological Activities of Ginsenoside Rd. Biomolecules.

[34] Shao X., Wang M., Wei X., Deng S., Fu N., Peng Q., Jiang Y., Ye L., Xie J., Lin Y. (2016). Peroxisome proliferator-activated receptor-γ: Master regulator of adipogenesis and obesity. Curr. Stem Cell Res. Ther..

[35] Gariballa S., Alkaabi J., Yasin J., Al Essa A. (2019). Total adiponectin in overweight and obese subjects and its response to visceral fat loss. BMC Endocr. Disord..

[36] Achari A.E., Jain S.K. (2017). Adiponectin, a Therapeutic Target for Obesity, Diabetes, and Endothelial Dysfunction. Int. J. Mol. Sci..

[37] Krause M.P., Milne K.J., Hawke T.J. (2019). Adiponectin-Consideration for its Role in Skeletal Muscle Health. Int. J. Mol. Sci..

