# Supplementary material for: Bioconverted Fruit Extract of Akebia Quinata Exhibits Anti-Obesity Effects in High-Fat Diet-Induced Obese Rats
Source: Nutrients. 2022 Nov 5;14(21):4683. doi: 10.3390/nu14214683 (PMC9656223; doi:10.3390/nu14214683)
Supplement: Supplementary file 1 [file nutrients-14-04683-s001.zip › nutrients-1955035-supplementary.pdf]

Supplementary Table S1. Plasma biochemical values

	ND	HFD	FE 150	BFE 150	FE 300	BFE 300
ALB (g/dL)	2.3±0.2	2.5±0.1	2.4±0.2	2.4±0.1	2.1±0.6	2.4±0.2
ALP (U/L)	569.2±64.5	609.8±96.2	536.1±0.2	481.9±156.8	472.9±106.4	574.0±138.9
AST (U/L)	106.0±15.4	135.7±29.5	85.8±28.0	102.8±37.6	75.5±28.6	99.5±19.3
BUN (mg/dl)	15.1±1.2	11.5±1.7	11.1±1.1	11.1±1.4	10.9±2.0	12.6±2.5
CRE (mg/dl)	0.5±0.1	0.4±0.1	0.4±0.0	0.4±0.0	0.5±0.1	0.4±0.1
GLU (mg/dl)	196.4±43.6	192.5±46.2	174.7±25.6	170.1±30.6	200.6±65.8	173.9±20.3
TP (g/dl)	6.2±0.5	6.8±0.3	6.3±0.6	6.8±0.1	5.7±1.5	6.4±0.5
HLD (mg/dl)	28.1±2.7	15.7±4.2	22.9±0.7	23.0±3.2	27.5±2.1	20.6±1.4
LDL (mg/dl)	6.4±0.6	7.9±1.0	10.7±0.9	10.0±1.1	11.9±0.8	8.1±0.1
